# The current status of cancer rehabilitation provided by palliative care units in Japan: a nationwide survey

**DOI:** 10.1186/s12885-025-13897-4

**Published:** 2025-03-13

**Authors:** Takuya Fukushima, Tetsuya Tsuji, Kazunori Takashima, Jiro Nakano

**Affiliations:** 1 Faculty of Rehabilitation, Kansai Medical University, 18-89 Uyamahigashicho, Hirakata, Osaka 573-1136 Japan; 2 Department of Rehabilitation Medicine, Keio University School of Medicine, Tokyo, Japan; 3 Department of Rehabilitation, Faculty of Health Sciences, Hiroshima Cosmopolitan University, Hiroshima, Japan

**Keywords:** Cancer rehabilitation, Palliative care unit, Advanced cancer, Evidence

## Abstract

**Background:**

To our knowledge, the current status of and barriers to cancer rehabilitation in palliative care units (PCUs) in Japan remain to be elucidated. If clarified, this information could help develop rehabilitation strategies to improve the quality of life of patients with cancer who need palliative care. Hence, this study aimed to clarify the current status of and barriers to cancer rehabilitation in PCUs in Japan by conducting a nationwide questionnaire survey.

**Methods:**

This nationwide questionnaire-based survey included 462 hospitals that met the facility criteria for PCU inpatient charges, and notified local health authorities. The questionnaire included questions on the implementation rate of cancer rehabilitation, the sufficiency/insufficiency of cancer rehabilitation and its reasons, and the reasons for and need not implement cancer rehabilitation in PCUs.

**Results:**

Among the 462 hospitals, we received responses from 265 hospitals (response rate: 57.4%). Cancer rehabilitation was implemented in 242 (91.3%) responding hospital PCUs. Among the implementing hospitals, 154 (63.6%) recognized cancer rehabilitation as inadequate. The main reasons for this insufficiency were ineligibility for medical fees for disease rehabilitation and a lack of rehabilitation staff. Among the non-implementing hospitals, the main reasons for not implementing cancer rehabilitation were ineligibility for medical fees for disease rehabilitation and a lack of rehabilitation staff. Of the non-implementation hospitals, 75.0% (*n* = 15) indicated the need for cancer rehabilitation in their PCUs.

**Conclusions:**

To ensure sufficient and quality-assured cancer rehabilitation in PCUs, it is necessary to allocate medical fees for disease rehabilitation, provide additional reimbursements, and increase the number of rehabilitation staff.

**Supplementary Information:**

The online version contains supplementary material available at 10.1186/s12885-025-13897-4.

## Background

Cancer rehabilitation can be applied at all stages of the disease, from prevention and functional recovery to maintenance of patients' function during their remaining time and in palliative care [1]. In the palliative phase, in particular, patients suffer from a wide range of unmet needs, such as gait-related difficulties, limitations in activities of daily living (ADL), physical and psychological symptoms, and reduced quality of life (QoL) [2, 3]. Furthermore, the families of patients with advanced cancer require education on physical care techniques and symptom management strategies [4]. Therefore, cancer rehabilitation is a crucial issue in palliative care.

Although palliative care professionals recognize the need for rehabilitation, it is provided inconsistently in palliative care units (PCUs), and a considerable proportion of patients worldwide may not receive the necessary rehabilitation services [5–7]. Previous research suggests that barriers to palliative rehabilitation include a lack of conceptual clarity and standardized tools to measure outcomes, as well as the need for additional education of rehabilitation staff in palliative cancer rehabilitation [8, 9]. Furthermore, culture, religious and spiritual beliefs, and ideological views markedly affect the needs and wishes of patients with cancer in the palliative phase [10, 11]. Therefore, the status of palliative care rehabilitation should be assessed on a country-by-country basis.

Regarding the status of PCUs in Japan, the first PCU in the country was established in 1981; it was later expanded to cover both cancer and acquired immune deficiency syndrome [12]. In the 1990s, a payment system for hospitalization in PCUs was established under the medical insurance system [12]. While this was a groundbreaking change because it provided a financial base for PCUs through national medical insurance, the daily medical fee was fixed at a certain amount regardless of treatment. Consequently, even if rehabilitation is performed in a PCU, it is impossible to bill reimbursement for the disease, and it is performed free of charge. This may have contributed to the decline in rehabilitation provisions and should be taken into consideration. In a previous study in Japan, Hasegawa et al. found that only 28.2% of patients received rehabilitation in a PCU [4]. While this is an important report that shows the current status of cancer rehabilitation in PCUs in Japan, it highlights the need for a nationwide survey of PCUs across Japan. To develop cancer rehabilitation in PCUs, it is necessary to understand the current status of these units in the country, considering the need to address various issues, including rehabilitation and healthcare systems. However, to the best of our knowledge, no study has elucidated the current status of and barriers to cancer rehabilitation in PCUs in Japan. If clarified, this information could be a keystone for developing rehabilitation strategies to improve the QoL of patients with cancer who need palliative care. Hence, this study was aimed at elucidating the current status of and barriers to cancer rehabilitation in PCUs in Japan by using a nationwide questionnaire.

## Methods

### Study design and population

This questionnaire-based nationwide survey was implemented in Japan and targeted 462 hospitals that met the facility criteria for inpatient charges for PCUs and have notified local health authorities. The questionnaire was sent to managers of the rehabilitation departments, including physical therapists (PT), occupational therapist (OT), speech and language therapist (ST), and physiatrists. Ethical approval was obtained from the institutional review board of Kansai Medical University (approval number: 2023418). All procedures in this study were conducted in accordance with the ethical standards of the Institutional Review Board, the national guidelines, and the 1964 Declaration of Helsinki and its later amendments. By completing the questionnaire, participants were considered to have provided informed consent.

### Survey method and questionnaire

The survey questionnaire was distributed by postal mail. In June 2024, the questionnaire was mailed to each eligible hospital, and questionnaire completion by a rehabilitation professional in a managing/supervisory position was requested. Hospitals that did not respond within 3 weeks of the initial mailing were sent a reminder. A team of physiatrists, PT, and OT specializing in cancer rehabilitation discussed and developed the survey questionnaire. The implementation status and sufficiency/insufficiency of cancer rehabilitation were surveyed. The reasons for insufficient cancer rehabilitation in the PCU were categorized as follows: ineligibility for medical fees for rehabilitation of diseases, lack of rehabilitation staff with the requisite knowledge/skills, lack of rehabilitation staff, not being referred to the rehabilitation unit, and inadequate facilities and equipment, among others. If rehabilitation was not provided in the PCU, the reasons given were ineligibility for medical fees for rehabilitation of diseases, lack of rehabilitation staff with the requisite knowledge/skills, lack of rehabilitation staff, not being referred to the rehabilitation unit, and inadequate facilities and equipment, among others. Additionally, for those facilities that had not yet provided rehabilitation, the need for rehabilitation in the PCU and the reasons for the need, if any, were surveyed in the following items: relief from physical symptoms, relief from psychological distress, maintain and improve physical function, maintain and improve ADL, maintain and improve QoL, relief from psychological distress and caregiving guidance for family members, and return to home support. If there was no need for rehabilitation, the reasons were surveyed using a 4-point scale (strongly agree, agree, disagree, and strongly disagree) in each of the following items: Lack of evidence for cancer rehabilitation in the PCU/palliative setting; No proof exists that cancer rehabilitation in the PCU is actually effective in relieving patients' physical and psychological symptoms; No proof exists that cancer rehabilitation in the PCU is actually effective in improving patients' physical function and ADLs; No proof exists that cancer rehabilitation in the PCU is actually effective in improving patients' QoL; No proof exists that cancer rehabilitation in the PCU is actually effective in relieving psychological distress and acquiring caregiving skills for family members; The details of the cancer rehabilitation program in the PCU are unknown; No rehabilitation staff available to provide cancer rehabilitation in the PCU; Lack of education about palliative care/palliative cancer rehabilitation programs; and Ineligibility for medical fees for rehabilitation of diseases in the PCU. Furthermore, information was obtained regarding hospital features, including the type of hospital (University hospital, cancer center, and general hospital, among others); the total number of beds (< 300, 301–600, 601–1000, and > 1001 beds); and the number of PCU beds (0, 1–10, 11–30, 31–60, and > 61 beds), rehabilitation staff (presence of physiatrists, actual number of PT, OT, and ST), and therapists who completed the Cancer Rehabilitation Educational Program for Rehabilitation (CAREER). CAREER is an educational system in Japan that trains rehabilitation professionals for the management of cancer [13, 14]. Participation in the workshop requires a group of several people, including one medical doctor, one nurse, and therapists per institution. It comprises an e-learning lecture followed by a 1-day case review and group work. The lectures provide basic knowledge on the evidence and practice of cancer rehabilitation, including palliative care. During the case review and group work, participants discuss the management and treatment of cancer-related conditions in small groups [1]. Details of the questionnaire are provided in Supplementary Table S1.

### Statistical analysis

Descriptive analysis was performed to determine the status of the implementation rate of cancer rehabilitation, sufficiency/insufficiency of cancer rehabilitation, reasons for and reasons for not implementing cancer rehabilitation in the PCU, and hospital characteristics. Data are expressed as the median value (interquartile range) or the number and percentage of participants and were analyzed using the IBM SPSS Statistics version 27 software (IBM SPSS, Chicago, IL, USA).

## Results

**Fig. 1:**
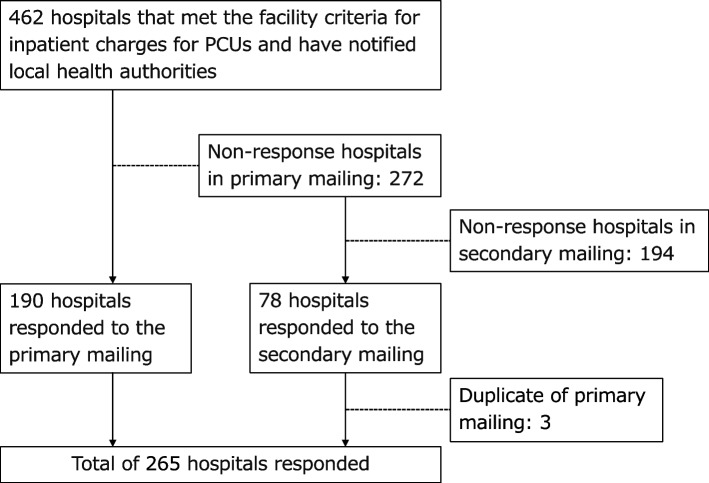
Flow diagram

Among the 462 hospitals that received the questionnaires, 265 responded (response rate: 57.4%), with 190 and 75 responses from the primary and secondary mailing, respectively (Fig. 1). PT was the most common respondents (*n* = 158; 59.6%). In terms of the characteristics of the hospitals from which responses were received, general hospitals were the most common hospital type (*n* = 241, 90.9%) and most hospitals had 11–30 PCU beds (*n* = 224, 84.5%). A detailed breakdown of the hospitals that responded according to facility and rehabilitation staff characteristics is shown in Table 1.

**Table 1:** Characteristics of facility and rehabilitation staff

Type of facilities	
University hospital	6 (2.3%)
Cancer center	10 (3.8%)
General hospital	241 (90.9%)
Other	4 (1.5%)
No answer	4 (1.5%)
Number of total beds	
<300 beds	118 (44.5%)
301–600 beds	118 (44.5%)
601–1000beds	25 (9.5%)
>1001beds	4 (1.5%)
Number of PCU beds	
0 bed	2 (0.8%)
1–10 beds	10 (3.8%)
11–30 beds	224 (84.5%)
31–60 beds	19 (7.2%)
>61beds	1 (0.4%)
No answer	9 (3.4%)
Existence of physiatrists	
Yes	98 (37.0%)
No	164 (61.9%)
No answer	3 (1.1%)
Number of rehabilitation staffs	
PT	16 [10–27]
OT	7 [4–14]
ST	4 [2–6]
Number of rehabilitation staffs who had completed the CAREER program	
PT	6 [1–11]
OT	3 [0–5]
ST	1 [0–3]

*CAREER* Cancer Rehabilitation Educational program for Rehabilitation teams, *OT* occupational therapists, *PCU* palliative care units, *PT* physical therapists, *ST* speech and language therapists

### Hospitals that implemented cancer rehabilitation in the PCU

We found that 91.3% (*n* = 242) of the responding hospitals implemented cancer rehabilitation in PCUs. All rehabilitation staff members, in order of PT, OT, and ST, were involved in cancer rehabilitation in the PCU, with concurrent positions being the most common. Among the hospitals that implemented cancer rehabilitation, 154 (63.6%) recognized that cancer rehabilitation in their PCUs was inadequate. The main reasons for this insufficiency were ineligibility for medical fees pertaining to disease rehabilitation (*n* = 133, 86.4%) and a lack of rehabilitation staff (*n* = 104, 67.5%) (Table 2).

**Table 2:** Implementation status of cancer rehabilitation in the PCU

Implementation status of cancer rehabilitation in the PCU	
Implementation	242 (91.3%)
Not implemented	20 (7.5%)
No answer	3 (1.1%)
Is cancer rehabilitation sufficiently implemented in the PCU?	
Sufficient	85 (35.1%)
Insufficient	154 (63.6%)
No answer	3 (1.2%)
Reasons for insufficient cancer rehabilitation in the PCU (multiple answers allowed)	
Ineligibility for medical fees for rehabilitation of diseases	133 (86.4%)
Lack of rehabilitation staff with the requisite knowledge/skills	30 (19.5%)
Lack of rehabilitation staff	104 (67.5%)
Not referred to rehabilitation unit	13 (8.4%)
Inadequate facilities and equipment	4 (2.6%)
Others	8 (5.2%)

*PCU* palliative care units

### Barriers to the implementation of cancer rehabilitation in PCUs

Regarding the hospitals that did not implement cancer rehabilitation in PCUs (*n* = 20, 7.5%), the main reasons for the lack of said implementation were ineligibility for medical fees for rehabilitation of diseases (*n* = 15, 75.0%) and a lack of rehabilitation staff (*n* = 11, 52.4%) (Table 3). Further, 75.0% of these hospitals (*n* = 15) indicated the need for cancer rehabilitation in PCUs. The main reasons for this response were relief from psychological distress (*n* = 12, 75.0%), maintenance and improvement of QoL (*n* = 7, 43.8%), relief from physical symptoms (*n* = 7, 43.8%), relief from psychological distress and caregiving guidance for family members (*n* = 6, 37.5%), and returning home support (*n* = 6, 37.5%) (Table 3). Regarding the hospitals that did not implement cancer rehabilitation in PCUs because they did not identify a need for cancer rehabilitation, the hospitals stated that they strongly agreed or agreed with the following reasons for not implementing cancer rehabilitation: lack of evidence for cancer rehabilitation in PCUs (*n* = 4, 80.0%); no definitive proof that cancer rehabilitation in PCUs is effective in relieving physical and psychological symptoms (*n* = 4, 80.0%), improving physical function and ADL (*n* = 5, 100.0%), improving QoL (*n* = 5, 100.0%), and relieving psychological distress and acquiring caregiving skills for family members (*n* = 3, 60.0%); not knowing the specific contents of cancer rehabilitation in PCUs (*n* = 3, 60.0%); lack of rehabilitation staffs (*n* = 5, 100.0%) and education on cancer rehabilitation in PCUs (*n* = 4, 80.0%); and ineligibility for medical fees for rehabilitation of diseases (*n* = 4, 80.0%) (Table 4).

**Table 3:** Reasons and need for cancer rehabilitation in the PCU

Reasons for not implementing cancer rehabilitation in the PCU (multiple answers allowed)	
Ineligibility for medical fees for rehabilitation of diseases	15 (75.0%)
Lack of rehabilitation staff with the requisite knowledge/skills	1 (4.8%)
Lack of rehabilitation staff	11 (52.4%)
Not referred to rehabilitation unit	1 (4.8%)
Inadequate facilities and equipment Others	2 (9.5%) 5 (23.8%)
Is there a need for cancer rehabilitation in the PCU?	
Yes	15 (75.0%)
No	5 (25.0%)
Reasons for the need for cancer rehabilitation in the PCU among the not implemented hospitals	
(Top 3 answers are allowed)	
Relief from physical symptoms	7 (43.8%)
Relief from psychological distress	12 (75.0%)
Maintenance and improve physical function	4 (25.0%)
Maintenance and improve ADL	5 (31.3%)
Maintenance and improve QoL	8 (50.0%)
Relief from psychological distress and caregiving guidance for family members	6 (37.5%)
Returning to home support	7 (43.8%)

*ADL* activities of daily living, *PCU* palliative care units, *QoL* quality of life

**Table 4:** Reasons for not identifying a need for cancer rehabilitation at non-implementing hospitals

	Strongly agree	Agree	Disagree	Strongly disagree
Lack of evidence for cancer rehabilitation in the PCU/palliative setting	0 (0.0%)	4 (80.0%)	1 (20.0%)	0 (0.0%)
There is no definitive proof that cancer rehabilitation in the PCU is effective in relieving patients' physical and psychological symptoms	0 (0.0%)	4 (80.0%)	1 (20.0%)	0 (0.0%)
There is no definitive proof that cancer rehabilitation in the PCU is effective in improving patients' physical function and ADLs	0 (0.0%)	5 (100.0%)	0 (0.0%)	0 (0.0%)
There is no definitive proof that cancer rehabilitation in the PCU is effective in improving patients' QoL	0 (0.0%)	5 (100.0%)	0 (0.0%)	0 (0.0%)
There is no definitive proof that cancer rehabilitation in the PCU is effective in relieving psychological distress and acquiring caregiving skills for family members	0 (0.0%)	3 (60.0%)	2 (40.0%)	0 (0.0%)
The details of the cancer rehabilitation program in the PCU are not known	0 (0.0%)	3 (60.0%)	2 (40.0%)	0 (0.0%)
Lack of rehabilitation staff available to provide cancer rehabilitation in the palliative care unit	2 (40.0%)	3 (60.0%)	0 (0.0%)	0 (0.0%)
Lack of education about palliative care/palliative cancer rehabilitation programs	1 (20.0%)	3 (60.0%)	1 (20.0%)	0 (0.0%)
Ineligibility for medical fees for rehabilitation of diseases in the PCU	2 (40.0%)	2 (40.0%)	1 (20.0%)	0 (0.0%)

*ADL* activities of daily living, *PCU* palliative care units, *QoL* quality of life

## Discussion

For the widespread development of cancer rehabilitation in PCUs in Japan, it is necessary to understand the current status of PCUs across the country from the perspective of medical facilities. Therefore, this nationwide questionnaire survey was conducted to clarify the current status of and barriers to cancer rehabilitation in PCUs in Japan. Our survey revealed that almost all (approximately 90%) hospitals in Japan have implemented cancer rehabilitation in their PCUs. However, through a questionnaire study of bereaved families, Hasegawa et al. revealed that only 28.2% of the patients underwent rehabilitation in PCUs [4]. This gap may arise from differences in the subjects of the surveys. Our survey showed that although many hospitals provided cancer rehabilitation in their PCUs, they may not have been able to provide it adequately to all patients who needed it. Approximately 60% of the hospitals regarded their cancer rehabilitation in PCUs as insufficient. The main reasons for insufficient cancer rehabilitation in PCUs were ineligibility for medical fees and a lack of rehabilitation staff. These were also the main reasons for not implementing cancer rehabilitation in PCUs. A similar situation exists for outpatient cancer rehabilitation in Japan, where the lack of reimbursement prevents manpower allocation to this area, resulting in inadequate therapeutic intervention [14]. To ensure adequate and quality-assured cancer rehabilitation in PCUs, efforts should be made to verify its effectiveness and establish evidence to apply for medical fees for disease rehabilitation and additional reimbursement. Subsequently, rehabilitation staff can be assigned to cancer rehabilitation in PCUs. In fact, a previous study identified human resources as key enablers of palliative care [15], supporting our direction. Additionally, although they do not appear to be the main factors in this survey, lack of rehabilitation staff with the requisite knowledge/skills, not being referred to the rehabilitation unit, and inadequate facilities and equipment were listed as contributing to insufficiency. Regarding staff training on therapists' knowledge and skills, a previous study identified it as a major barrier to implementing palliative care in cancer practice [15]. There is a gap between this study and previous studies regarding whether lack of requisite knowledge/skills was the main factor that may be influenced by cancer rehabilitation education in Japan. Japan's cancer rehabilitation education system comprises the CAREER program, which provides a series of workshops to train cancer rehabilitation specialists [13, 14], ensuring the knowledge and skills of the staff involved in cancer rehabilitation. A previous study has identified communication among healthcare providers and systems building as facilitators of palliative care [15]. These factors from the previous study align with the barriers identified in this study of not being referred to rehabilitation units and inadequate facilities and equipment, underscoring the importance of addressing these factors.

According to the results of this study, approximately 75% of hospitals that did not implement cancer rehabilitation recognized the need for cancer rehabilitation in PCUs. Furthermore, the rehabilitation staff at these hospitals recognized the need for cancer rehabilitation for relief from physical and psychological distress, maintenance and improvement of QoL, caregiving guidance for family members, and support for returning home, as has been reported previously as well [16, 17]. This suggests that despite not implementing cancer rehabilitation, rehabilitation staff recognize the need for and the clinical efficacy of cancer rehabilitation in PCUs, which supports the effectiveness of the CAREER program.

A comprehensive view of the results of this study suggests that evidence building is a common issue in recognizing the need for cancer rehabilitation in PCUs and disseminating its quality-assured development. Although there are some studies indicating the efficacy of palliative cancer rehabilitation [16, 18–21], the evidence and the recommendations in the guidelines are not strong [22]. Clinically, when providing cancer rehabilitation in PCUs, it is important to approach each case with sincerity, document evaluations and interventions objectively, and verify their effectiveness. Furthermore, culture and other factors influence outcomes in palliative care. Therefore, Japan should conduct original clinical research to provide relevant evidence. This could improve cancer rehabilitation by allocating reimbursements, additional fees, and rehabilitation staff to PCUs. Addressing these issues will be a challenge for the future. Furthermore, sharing these results with various professions, raising awareness of the need for and effectiveness of cancer rehabilitation in PCUs, and educating staff are suggested as key strategies.

This study had some limitations. First, the study does not reflect the situation in all PCUs in Japan, which is a subject for future research. Second, the study was limited to cancer rehabilitation in PCUs in Japan, which limits its generalizability to other countries and diseases. Third, although a team of physiatrists, PT, and OT specializing in cancer rehabilitation discussed and developed the survey questionnaire, we could not validate the effectiveness of the questionnaire before the start of the study. Fourth, although the response rate was approximately 60%, further follow-up by phone or web form could have improved it. Finally, response bias may have affected this study’s results. To avoid this, we implemented the reminder mailing to reduce selection and non-response biases. Additionally, we attempted to reduce leading and ambiguous phrases in the wording of the questionnaires.

## Conclusion

Cancer rehabilitation has been implemented in almost all hospitals with PCUs in Japan. However, approximately 60% of these hospitals regarded cancer rehabilitation in their PCUs as insufficient, with ineligibility for medical fees and a lack of rehabilitation staff being the main contributing factors. To expand and ensure sufficient and quality-assured cancer rehabilitation in PCUs, efforts should be made to verify the effectiveness of said cancer rehabilitation and establish stronger evidence. These efforts could improve cancer rehabilitation by allocating medical fees for specialized rehabilitation, additional reimbursements, and dedicated rehabilitation staff to PCUs.

## Supplementary Information

Supplementary Material 1.

## Data Availability

The data that support the findings of this study are available from the corresponding author upon reasonable request.

## Abbreviations

ADL: Activities of daily living
CAREER: Cancer Rehabilitation Educational Program for Rehabilitation
OT: Occupational therapist
PCUs: Palliative care units
PT: Physical therapists
QoL: Quality of life
ST: Speech and language therapist
