# Supplementary material for: The current status of cancer rehabilitation provided by palliative care units in Japan: a nationwide survey
Source: BMC Cancer. 2025 Mar 13;25:451. doi: 10.1186/s12885-025-13897-4 (PMC11905477; doi:10.1186/s12885-025-13897-4)
Supplement: Supplementary file 1 — Supplementary Material 1. [file 12885_2025_13897_MOESM1_ESM.docx]

Supplementary Table S1. Details of questionnaire

| Questionnaire | Contents | Choices |
| --- | --- | --- |
| 1. Facilities overview | 1-a. Type of facilities | University hospital, cancer center, general hospital, other |
|  | 1-b. Number of total beds | <300 beds, 301–600 beds, 601–1000 beds, >1001beds~ |
|  | 1-c. Number of PCU beds | 0 bed, 1–10 beds, 11–30 beds, 31–60 beds, >61 beds |
|  | 1-d. Presence of physiatrists | Yes/No |
|  | 1-e. Number of rehabilitation staffs (PT, OT, ST) | Actual number |
|  | 1-f. Number of rehabilitation staffs who had completed the CAREER program (PT, OT, ST) | Actual number |
| 1. Implementation of cancer rehabilitation in the PCU | 2-a. Does your hospital provide cancer rehabilitation in the PCU? | Yes/No |
|  | 2-b. If 2-a is yes, is cancer rehabilitation in the PCU sufficiently implemented? | Sufficient/Insufficient |
|  | 2-c. If 2-b is insufficient, what are the reasons for insufficient cancer rehabilitation in the PCU? | Ineligibility for medical fees for rehabilitation of diseases, lack of rehabilitation staffs with the requisite knowledge/skills, lack of rehabilitation staffs, not referred to rehabilitation unit, inadequate facilities and equipment, others (multiple answers allowed) |
|  | 2-d. If 2-a is no, what are the reasons for not implementing cancer rehabilitation in the PCU? | Ineligibility for medical fees for rehabilitation of diseases, lack of rehabilitation staffs with the requisite knowledge/skills, lack of rehabilitation staffs, not referred to rehabilitation unit, inadequate facilities and equipment, others (multiple answers allowed) |
|  | 2-e. If 2-a is no, is there a need for cancer rehabilitation in the PCU? | Yes/No |
|  | 2-f. If 2-e is yes, what are the reasons for the need for cancer rehabilitation in the PCU? | Relief from physical symptoms, relief from psychological distress, maintain and improve physical function, maintain and improve ADL, maintain and improve QoL, relief from psychological distress and caregiving guidance for family members, return to home support (Top 3 answers are allowed) |
|  | 2-g If 2-e is no, the following questions were asked about why there is no need for cancer rehabilitation in the PCU.   - Lack of evidence for cancer rehabilitation in the PCU/palliative setting. - There is no proof that cancer rehabilitation in the PCU is actually effective in relieving patients' physical and psychological symptoms. - There is no proof that cancer rehabilitation in the PCU is actually effective in improving patients' physical function and ADLs. - There is no proof that cancer rehabilitation in the PCU is actually effective in improving patients' QoL. - There is no proof that cancer rehabilitation in the PCU is actually effective in relieving psychological distress and acquiring caregiving skills for family members. - The details of the cancer rehabilitation program in the PCU are not known. - No rehabilitation staff available to provide cancer rehabilitation in the palliative care unit. - Lack of education about palliative care/palliative cancer rehabilitation programs - Ineligibility for medical fees for rehabilitation of diseases in the PCU | Strongly agree, agree, disagree, strongly disagree, no answer |

ADL, activities of daily living; CAREER, Cancer Rehabilitation Educational program for Rehabilitation teams; OT, occupational therapists; PCU, palliative care units; PT, physical therapists; QoL, quality of life; ST, speech and language. therapists.
